# BMP2 response pattern in human lung fibroblasts predicts outcome in lung adenocarcinomas

**DOI:** 10.1186/s12920-015-0090-4

**Published:** 2015-04-29

**Authors:** Michal Rajski, Annika Saaf, Martin Buess

**Affiliations:** Department of Biomedicine, University of Basel, Hebelstrasse 20, CH-4031 Basel, Switzerland; Department of Biochemistry and Molecular Biology, The University of Chicago, 900 East 57th Street, Chicago, IL 60637 USA; Division of Medical Oncology, Department of Internal Medicine, St. Claraspital, Kleinriehenstrasse 20, CH-4016 Basel, Switzerland; Institute of Physiology, University of Zürich, Winterthurerstrasse 190, CH-8057 Zürich, Switzerland

## Abstract

**Background:**

Bone morphogenetic proteins play important roles in development, morphogenesis and cancer. With this study we aimed to characterize the response of lung stromal fibroblasts to BMPs and their antagonists on a genome wide level and investigate its potential role in human lung adenocarcinomas.

**Methods:**

We used an *ex vivo* culture model and measured gene expression changes in human lung fibroblasts after stimulation with BMPs and their antagonists using HEEBO microarrays. The *in vitro* data were correlated with *in vivo* observations in published expression datasets of human lung adenocarcinomas.

**Results:**

We have systematically analyzed the response to BMP2, BMP4, BMP7 and their antagonists, Gremlin and Noggin, to define common and specific gene expression patterns. A BMP2 induced gene expression signature was defined, which is specific for stromal fibroblasts. Gene expression profiles from lung adenocarcinoma biopsies were analyzed to determine the prognostic significance of the “Fibroblast specific BMP2 induced gene list”. This gene list successfully segregated patients with different prognostic outcome in 3 datasets. In a small dataset (Garber et al.) there was a strong trend for a worse prognosis of patients with adenocarcinomas of all stages over-expressing the “Fibroblast specific BMP2 induced gene list”. In two larger datasets with stage I adenocarcinomas we observed a significantly worse disease-free (p = 0.002, Lee et al. and p = 0.002, Bhattacharjee et al.) and overall survival (p = 0.0002).

**Conclusions:**

The effects of BMPs and their antagonists are heterogeneous in different cell types. The gene expression pattern induced by BMP2 in primary lung fibroblasts may predict outcomes of patients with lung adenocarcinomas.

**Electronic supplementary material:**

The online version of this article (doi:10.1186/s12920-015-0090-4) contains supplementary material, which is available to authorized users.

## Background

The bone morphogenetic proteins (BMPs) are secreted signaling molecules that play important roles in numerous aspects of metazoan development, including stem cell regulation, fate determination, body axis specification, cell polarity, proliferation and differentiation [1]. Thus, alterations might play a role in cancer.

In human lung cancer, BMP2 has been shown to be over-expressed [2]. It activates Smad1/5, thereby increasing Id1 expression and promoting invasion in lung cancer cells [3].

It has long been established that epithelial-mesenchymal interactions are essential for development and organogenesis [4] such as the skin, teeth, gut, and lungs. More recently, it has been recognized that cancer is a tissue-based disease of stromal cells co-evolving with carcinoma cells. Increasing evidence suggests that tumorigenesis is dependent upon contextual signals received from the stroma [5] and epithelial-mesenchymal interactions are playing an important role in the initiation and progression of tumorigenesis. As the tumor develops, the surrounding stroma provides tumor cells with growth factors and favorable matrix components that foster proliferation, migration and colonization of distant organs. In fact, the reciprocal interaction of stromal cells and carcinoma cells drives carcinogenesis. The most abundant mesenchymal cells in the stroma of most tissues and organs are fibroblasts. These cells secrete extracellular matrix components and signaling molecules that contribute to the establishment of customized microenvironments for epithelial cells and provide a specialized niche for tissue stem cells [6]. Dissecting the molecular mechanisms that underlie cell-to-cell signaling and crosstalk between epithelial cells, stem cells, fibroblasts and other stromal cells in tissue microenvironments is essential to understand normal development and carcinogenesis [7]. Here, we focus on one molecular element that may play a role in epithelial-mesenchymal interactions in development and cancer – the transcriptional response of fibroblasts to BMP-induced signals. Given the multifaceted roles of BMP signaling in these processes [8], its transcriptional effects on fibroblastic stromal cells are of great interest. However, in lung cancer, the genome-wide effects of the involved signaling molecules on stromal fibroblasts have not yet been systematically described. Therefore, to build a systematic foundation of knowledge, we focused on the effects of BMPs and their antagonists, Gremlin and Noggin, on transcription in normal human lung fibroblasts. Importantly, we also demonstrate that the BMP2 gene signature identified in the lung fibroblasts *in vitro* has significant prognostic relevance in human lung adenocarcinomas and may thus serve as novel prognostic cancer markers and therapeutic targets in future.

## Methods

### Cell culture

Primary human fibroblasts (CCL-171) and the human breast cancer cell lines MDA-MB-231 and T47D were obtained from the American Type Culture Collection (ATTC, Atlanta, Georgia, USA) on 2005 November 15. After resuscitation the cells were propagated in Dulbecco’s modified Eagle’s medium (D-MEM, Invitrogen, Carlsbad, USA) supplemented with 10% heat-inactivated FBS (Invitrogen, Carlsbad, CA, USA), 4.5 g/l glucose, 4 mM L-glutamine, 100 U/ml penicillin and 100 μg/ml streptomycin (Gibco, Carlsbad, CA, USA). The cells were maintained by regular passages when confluence was reached and used for the experiments within 3–4 months. The study was approved by the Ethikkommission beider Basel, Switzerland (approval No. 271/05).

### BMP stimulation experiments

For the experiment, 30’000 cells/cm^2^ were seeded in 3 ml of 5% FBS D-MEM and incubated for 6 h to allow attachment. The cells were washed extensively with phosphate-buffered saline and starved for 48 h in fresh low-serum D-MEM supplemented with 0.2% FBS. The cells were starved to reduce the effects of any stimulation from the regular cell culture medium. The medium was subsequently replaced with fresh low-serum D-MEM with or without 200 ng/ml BMP2 (human recombinant in Escherichia coli; Sigma Aldrich, St. Louis, MO, USA), 24 ng/ml BMP4, 200 ng/ml BMP7, 240 ng/ml Noggin or 1 μg/ml Gremlin. The cells were stimulated for 24 h, and the RNA was harvested to test the effects of BMP and their antagonists on mRNA expression patterns.

### RNA extraction and amplification

After aspirating the culture medium, the cell monolayer was washed once with phosphate-buffered saline. The cells were lysed in a buffer containing guanidine isothiocyanate (RLT buffer, QIAGEN, CA, USA). The total RNA was extracted with an RNeasy kit (QIAGEN, Valencia, CA, USA) according to the manufacturer’s instructions. The RNA concentration was measured with a NanoDrop system spectrophotometer (ND-1000 Spectrophotometer Technologies, Wilmington, NC, USA). The integrity of extracted RNA was assessed by electrophoresis in a 1% agarose gel in MOPS buffer. For mRNA amplification, an Amino Allyl MessageAmp™ II aRNA Amplification Kit was used (Ambion, Austin, TX, USA). The amplification of mRNA from 500 ng total RNA, purification of the cDNA, in vitro transcription and purification of aRNA were performed according to the manufacturer’s instructions. The integrity and quantity of the amplified RNA were verified as described above.

### Gene expression analysis with HEEBO arrays

Global gene expression was assessed using the Human Exonic Evidence Based Oligonucleotide (HEEBO) microarrays produced at the Stanford Functional Genomic Facility (Stanford, CA, USA). The HEEBO microarrays consisted of 44,544 70-mer probes, which included the following: (a) constitutive exonic probes (30,718); (b) alternatively spliced/skipped exonic probes (8,441); (c) non-coding RNA probes (196); (d) BCR/TCR Genic/regional probes (372); (e) other probes (843); and (f) controls.

The complete details regarding the clones on the arrays can be found at the Stanford functional genomics facility website (https://microarray.org/sfgf/). For the microarray experiments, 8 μg of amplified RNA (aRNA) was mixed with the doping controls. The samples were vacuum dried, resolved in coupling buffer and labeled with Cy5 dye. The labeled samples were pooled with equal amounts of reverse color Cy3-labeled amplified reference RNA from Stratagene (Stratagene, Santa Rosa, CA, USA). The labeled aRNA was purified using an AminoAllyl MessageAmp™ II aRNA Amplification Kit (Ambion) according to the manufacturer’s instructions and fragmented using fragmentation reagents (Ambion). The fragmented probe was added to a hybridization buffer containing Cot/PolyA/tRNA (0.05 μg/uL each), 0.3% SDS and 3.3 × SSC supplemented with HEPES buffer. Following a denaturing step at 100° C, the probe was placed on the microarray for competitive hybridization. After 18 h, the slides with hybridized probe were sequentially washed, immediately dried in an ozone-free environment and scanned using an Axon Scanner 4100A (Axon Instruments, CA, USA). The data analysis and clustering Microarray fluorescent image analysis were performed using the software Genepix Pro version 5.0 3.0.6.89 (Axon Instruments, Union City, CA, USA). Any spots with obvious array artifacts or poor technical quality were manually removed from any further analysis. The raw data files were stored in the Stanford Microarray Database-Princeton University and are freely available (http://smd.princeton.edu/cgi-bin/publication/viewPublication.pl?pub_no=1095) [9] as well as in NCBI’s Gene Expression Omnibus and are accessible through GEO Series accession number GSE 66142 (http://www.ncbi.nlm.nih.gov/geo/).

### Data analysis

The data were expressed as the log2 ratio of the fluorescence intensity of the sample and fluorescence intensity of the reference for each element on the array. A sequential data filtering procedure was applied to include only measurements fulfilling our quality requirements (i.e. data with a Cy3 channel or Cy5 channel mean intensity over median background intensity greater than 1.5 and a Pearson regression correlation of the pixels within the spot of greater than 0.6). The genes that did not meet these criteria for at least 80% of the measurements across the experimental samples were excluded from further analysis. The data were evaluated by unsupervised hierarchical clustering with the Cluster® software using Pearson correlation (non-centered metric) and average linkage and displayed using TreeView® software [10].

### SAM analysis

SAM is a statistical approach to identify genes with expression patterns that are significantly associated with specific characteristics of the sample sets [11]. The Excel-SAM-package version 2.1. was obtained from the webpage of the Tibshirani Lab: http://statweb.stanford.edu/~tibs/SAM/. SAM analysis was applied to the BMP dataset to examine differences between stimulated and unstimulated cells and between cells exposed to different stimuli. A two-way, unpaired test or a multiclass test was performed to compare the two groups of interest. A 10-nearest neighbor imputation engine was applied to estimate missing data [12], and 100 permutations were performed to compute the expected values and calibrate false positives. The average amount of imputed values was 5.17 +/− 4.56 percent.

### Correlation of gene expression changes upon exposure to different stimuli

To test for similarities between the expression changes of single genes upon stimulation with BMP2, BMP4, BMP7, Noggin and Gremlin we calculated the Pearson correlation coefficient using the R software (R Development Core Team. A language and environment for statistical computing. Available: http://www.R-project.org) and displayed the results as scatter plots.

### Human cancer datasets

The dataset published by Garber and colleagues [13] contains global gene expression profiles for 67 human lung cancers derived from 56 patients. 41 of them were adenocarcinomas with survival data for 24 patients.

The dataset published by Lee and colleagues [14] contains global gene expression profiles of 138 human lung cancers, of which 63 were adenocarcinomas associated with survival data. The dataset published by Bhattacharjee [15] contains mRNA expression levels of 12,600 transcript sequences from 186 lung tumor samples, including 139 adenocarcinomas. Of these, 125 samples were associated with clinical data (some patients were in multiple runs). The Bhattacharjee dataset was obtained from the Broad Institute website, and the Garber dataset was acquired from the SMD webpage [9]. A list of 156 unique clones comprising 67 genes building the “Fibroblast specific BMP2 induced gene list” was extracted from the three datasets. To avoid possible overweighting of clones from Unigene clusters that matched more than one probe on the array, the expression values derived from probes matched to the same Unigene cluster were averaged. Only genes that had greater than 80% data values and tumor samples from patients having complete clinical data were used. The resulting dataset was subjected to average linkage unsupervised clustering, and the results were displayed using TreeView software [10]. All statistical tests were performed using the R statistical software (version 2.10.1). Survival curves were obtained using the Kaplan-Meier estimator and univariate Cox proportional hazards regression models were fitted (R package “survival”) R Development Core Team. A language and environment for statistical computing. Available: http://www.R-project.org. Accessed 2013 December 2. Hazard ratios and cox p-values are given to all the curves.

### Continuous scoring

The patients in the Lee [14] and the Bhattacharjee datasets [15] were stratified based on a continuous score derived from the “Fibroblast specific BMP2 induced gene list”, as described previously. Briefly, the average expression level of the genes in the “Fibroblast specific BMP2 induced gene list” was calculated for each patient attributing a score. The patients were then divided into two groups, separating them by the median value of the continuous scores. Only patients with stage I cancers were included in the survival analysis. Overall survival was based on the number of deaths from any cause, and the patients were censored at the final follow up. Disease-specific survival analysis was based on the number of deaths caused by the disease, with patients censored at the last follow-up. In this analysis, patients who died from other causes were considered alive and censored. The survival statistics were calculated as described above.

### GO::TermFinder analysis

GO::TermFinder uses a list of genes as input and determines whether those genes have gene ontology (GO) terms overrepresented in their combined set of annotations compared with what would be expected by chance for a randomly selected group of genes from the background population of all of the genes [16]. In our analysis, to calculate the frequency of particular annotations we used the gene lists from specific clusters and the full parental gene lists from the same heatmap as a background file. For a SAM-derived signature, we used an input file for SAM analysis as the gene list. For p-value correction we used the Bonferroni correction method, as described by Boyle et al. [16].

## Results

### BMP2 stimulation of lung stromal fibroblasts induces regulatory genes of the BMP signaling pathway

**Figure 1:**
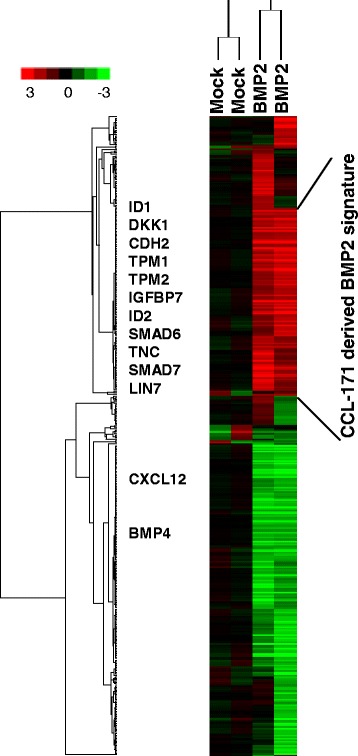
Response of lung stromal fibroblasts (CCL-171) to BMP2 stimulation. Biologically independent duplicate samples of non-stimulated (Mock) and BMP2 stimulated CCL-171 cells for 24h were profiled for gene expression changes using HEEBO- microarrays. After filtering for data quality as described in the methods section, filtering for data distribution (SD > =0.7) and zero-transformation by the mean of the non-stimulated samples unsupervised hierarchical clustering was performed and the data were displayed in a heatmap. The genes are presented in rows and experiments in columns. Red and green correspond to up- and down-regulation, respectively. The intensity of the color reflects the magnitude of the change in the expression level as depicted on a color key. The genes of interest and the CCL-171 derived BMP2 signature are indicated.

The aim of this study was to uncover the transcriptional responses to BMP signaling in lung fibroblastic stroma. To characterize the effects of BMP2 on stromal cells, we stimulated pre-starved primary human fibroblasts (CCL-171) with BMP2 in a physiological concentration of 200ng/ml for 24 h. Biologically independent duplicate samples were profiled for gene expression changes using human exonic evidence-based oligonucleotide (HEEBO) microarrays. After filtering for data quality as described in the methods section, filtering for data distribution (SD > =0.7) and zero-transformation by the mean of the duplicate mock control average linkage unsupervised hierarchical clustering (Pearson correlation) was performed and the data were presented in a heatmap (Figure 1). Following BMP2 stimulation, we observed a remarkable change in the gene expression profile. 171 genes exhibited a greater than 1.5-fold increase (mean: 2.68, standard deviation: 0.86 ) and 206 genes showed a more than 1.5-fold decrease in the expression level (mean decrease: 2.99, standard deviation: 1.604) (Additional file 1: Table S1).

A notable feature of the transcriptional response was the induction of genes with direct roles in the regulation of BMP signaling either as inhibitors (e.g., BAMBI) or positive regulators (e.g., BMPR1A). Co-expression of both negative and positive regulators of BMP signaling provides a network of opposing control mechanisms that are likely to contribute to the robust and precise regulation of BMP target gene expression. Furthermore, we detected an up-regulation of BMP target genes such as SMAD7, ID1, ID2 and ID3. Other genes such as BMP4 and CXCL12 were systematically down-regulated. The down-regulation of CXCL12 indicates a tie to stem cell regulation [17]. The “CCL-171-derived BMP2” signature as marked in Figure 1 (Additional file 2: Table S2), also contains genes that are known to be involved in developmental processes, including GATA6, DKK1, ID2, BAMBI, SERPINE1, and PTGIS.

The identification of transcription factors and other regulatory genes involved in the transcriptional response (e.g. HEY1, PHTF2, GATA6, and LMCD1) suggests that a more extensive reprogramming of transcription occurs downstream of the primary target genes; the mechanism of target gene activation is likely to be far more complex than direct activation through SMADs, involving a cascade of downstream transcription factors and regulatory proteins that play a role in further regulation of secondary BMP2 target genes. Among the transcription factors regulated by BMP signal transduction, it was interesting to find HEY1 and GATA6, which play important roles in pattern formation, morphogenesis and body-axis specification. The increased expression of TPM1, TPM2 and TNC [18] indicates a fundamental reprogramming of normal lung fibroblasts such that they exhibited a gene expression pattern that is typically found in carcinoma-associated fibroblasts.

For an unbiased assessment of which features are shared by members of the “CCL-171-derived BMP2” signature and to verify the significance of enrichment of a specific gene ontology, we used the GO::TermFinder tool [16]. The analysis revealed that the “CCL-171-derived BMP2” signature as shown in Figure 1 (Additional file 2: Table S2) is significantly enriched for genes involved in biological processes such as the cellular response to chemical stimuli and the BMP signaling pathway with Bonferoni corrected p-values of 0.00017 and 0.006, respectively (Additional file 3: Table S3).

Furthermore to identify genes with a significant change in expression levels we performed a two class unpaired SAM analysis using a false discovery rate <1%. 76 significantly induced and 151 significantly repressed genes after BMP2 stimulation are shown in Additional file 4: Table S4. According to our expectations, all the significantly up-regulated genes are contained within the list of Additional file 1: Table S1. In addition 10 genes not comprised within Additional file 1: Table S1 were found to be significantly down-regulated with SAM analysis, namely: GNG11, ABHD5, PHF17, GAS1, ARSI, JUN, KLHL29, CHEK2, ST6GAL1, PDGFRA, H1F0, BTG1 (fold changes of these genes: 2.15-2.58 fold decrease).

### A global picture of genes that are differentially expressed in response to BMP stimulation or inhibition in lung stromal fibroblasts

**Figure 2:**
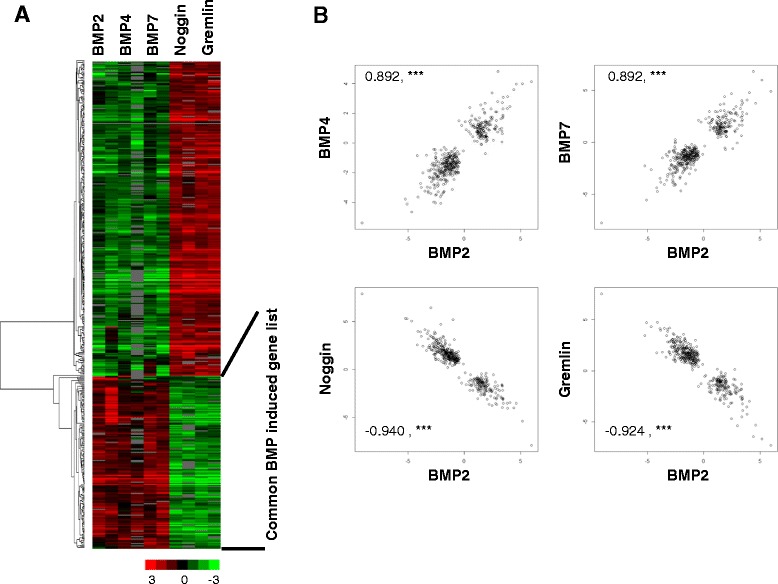
Effects of BMP 2, 4 and 7 and gremlin and noggin on CCL-171 cells. **A)** A multiclass SAM analysis was performed to select for genes that are differentially expressed in response to BMP2, BMP4, BMP7, Noggin or Gremlin using a FDR of less than 1% as the cut-off value. The selected genes were organized by unsupervised hierarchical clustering and gene expression changes plotted in a heatmap. The legend bar maps color versus the magnitude of changes in transcript levels relative to the mock-treatment level. The “common BMP induced gene list” is indicated, and the genes included in the list are shown in Additional file 5: Table S5. **B)** Scatter plots demonstrating the correlation of expression changes of all genes identified by SAM upon stimulation with BMP2 (x-axis) versus BMP4, BMP7, Noggin and Gremlin( y-axis). The Pearson correlation coefficient r is given. ***corresponds to a p-value < 0.001.

To obtain a more general picture of the transcriptional responses to BMP signaling, we examined the gene expression changes in response to physiological concentrations of BMP2, BMP4, and BMP7, as well as their antagonists, Noggin and Gremlin, in CCL-171 cells. CCL-171 cells were starved, and the medium was subsequently replaced with fresh low-serum D-MEM with or without 200 ng/ml BMP2 (human recombinant in Escherichia coli; Sigma Aldrich), 24 ng/ml BMP4, 200 ng/ml BMP7, 240 ng/ml Noggin, or 1 μg/ml Gremlin*.* To search for genes with a significant change in expression upon exposure to at least one of the agents, we performed a multiclass SAM analysis on BMP2, BMP4, BMP7, Noggin, Gremlin and mock stimulated biologically independent duplicate samples of CCL-171 with a false discovery rate of 1% [11] After zero-transformation with the mean expression levels of the mock stimulated samples we performed unsupervised hierarchical clustering of the genes identified by SAM and displayed the data as a heatmap (Figure 2A). A set of 115 genes was identified that were commonly induced by BMP2, BMP4 and BMP7 (Additional file 5: Table S5). To correlate the responses of all genes derived by SAM upon BMP2 stimulation with the responses to BMP4, BMP7, Noggin and Gremlin we calculated the Pearson correlation coefficient r and displayed their correlation as a scatter plot (Figure 2B). Thus the response to BMP2 observed in CCL-171 cells was highly significantly correlated to that observed in response to BMP4 (r = 0.892, p < 0.001) and BMP7 (r = 0.892, p < 0.001). An expression pattern opposing the BMP responses were observed in response to Noggin (r = −0.940, p < 0.001) and Gremlin (r = −0.924, p < 0.001), as would be expected for antagonists.

### A comparison of the effect of BMP2 stimulation in lung stromal fibroblasts and breast cancer cells

To determine whether the response to BMP2 is a general effect or specific for mesenchymal cells such as the lung fibroblasts (CCL-171), we compared the gene expression profile of BMP2-stimulated CCL-171 cells with the expression profiles of the breast cancer cells MDA-MB-231 and T47D treated with BMP2. First, to systematically define a gene signature reflecting the common response to BMP2 shared by CCL-171, MDA-MB-231 and T47D cells, we used a two class SAM with block permutation to identify genes with a significant change using a false discovery rate of less than 0.8%. A common set of 80 genes that were significantly induced, and 200 genes that were significantly repressed in all three cell types were identified (Additional file 6: Table S6).

**Figure 3:**
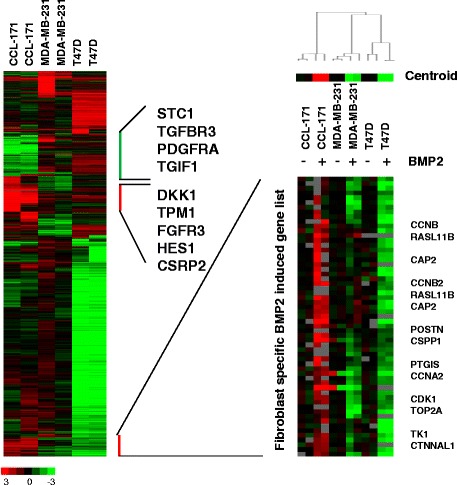
Effects of BMP2 stimulation on CCL-171, MDA-MB321 and T47D cells. Biologically independent duplicate samples of CCL-171, MDA-MB-231 and T4D7 cells were exposed to BMP2 for 24 h. To determine the cell type specific gene expression changes, the mean of the mock gene expression profile was subtracted from mean of the gene expression profiles of the duplicates of the respective cell type induced by BMP2 for CCL-171, MDA-MB-231 and T47D. The profiles of induced or repressed genes were merged. Genes with a difference in expression of at least 4 fold were displayed after unsupervised hierarchical clustering in a heatmap. A set of genes specifically up-regulated in CCL-171, representing the “fibroblast specific BMP2 induced gene list” is indicated. In the zoomed image the genes of the “fibroblast specific BMP2 induced gene list” are shown for all duplicate samples. The array tree determined by unsupervised hierarchical clustering using average linkage of all the genes of the arrays is shown to demonstrate consistency of the duplicates. The mean expression values of all the genes of the “fibroblast specific BMP2 induced gene list” is shown above the cluster of all genes as expression level of the centroid.

Second, we intended to determine the cell type specific gene expression changes upon BMP2 stimulation. The gene expression levels of CCL-171, MDA-MB-231 and T47D upon stimulation with BMP2 were subtracted by the mean expression levels of duplicate samples of the same cell types with mock stimulation. Genes with a difference in expression of at least 4 fold were selected and after unsupervised hierarchical clustering displayed in a heatmap (Figure 3). We observed a cell type dependent induction or repression of specific genes. We observed genes that were specifically up- or down-regulated in CCL-171 cells, including POSTN, a gene that was recently described to be essential for the formation of metastatic stem cell niches [19]. The fibroblast-specific up-regulation of topoisomerases and cyclins indicates an induction of fibroblast proliferation in response to BMP2 (Figure 3). A set of 67 genes, which are specifically induced in CCL-171 compared to MDA-MB-231 and T47D is referred as the “Fibroblast specific BMP2 induced gene list” (Additional file 7: Table S7). To support the up-regulation of this cluster in BMP2 stimulated CCL171 cells compared to non-stimulated CCL-171 cells and compared to MDA-MB-231 and T47D we calculated the average expression levels of the genes in the cluster (centroid) for each cell line and condition. A graphic representation of the average expression value is shown as centroid above the heatmap of the magnified area in Figure 3.

### BMP4 stimulation of lung stromal fibroblasts induces a time-dependent gene expression signature

**Figure 4:**
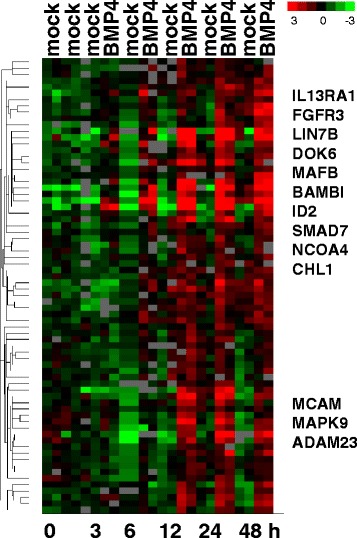
Time course analysis of BMP4 stimulation in CCL-171 cells. Biologically independent duplicate samples of CCL-171 cells were exposed to 24 ng/ml BMP4 for 1, 3, 6, 12, or 24 h in parallel with a mock control. Data were analyzed by unsupervised hierarchical clustering and plotted as a heatmap. A cluster of genes induced by BMP4 over time is shown and genes of interest are marked.

To exemplarily show the development of the gene expression changes in response to BMP stimulation over time we performed a 48 h time course analysis after BMP4 stimulation in CCL-171 cells. CCL-171 cells were first placed under replicative quiescence in DMEM media with 0.1% serum for 48 h and then exposed to fully supplemented media, DMEM with 10% serum, and incubated with BMP4 for 1, 3, 6, 12, 24 or 48 h. The temporal effect of BMP4 on gene expression was assessed using DNA microarrays. The BMP4 dependent changes in the gene expression profiles over time were assessed by normalization against the time dependent changes with mock stimulation and normalization against the gene expression profile at time 0. Some genes were up-regulated as early as 3 hours, whereas others were only expressed after 24 hours of stimulation with BMP4 (Figure 4). The strong and highly significant correlation (r = 0.892, p < 0.001) of the gene expression changes upon BMP4 and BMP2 stimulation over 24h (Figure 2B) suggests a concordant and reliable pattern of gene expression changes in CCL-171 upon 24h of stimulation by BMP4 and BMP2. This suggests the response to BMP2 to be suitable for further analysis.

### Prognostic significance of the “Fibroblast specific BMP2 induced gene list” in human lung adenocarcinomas

**Figure 5:**
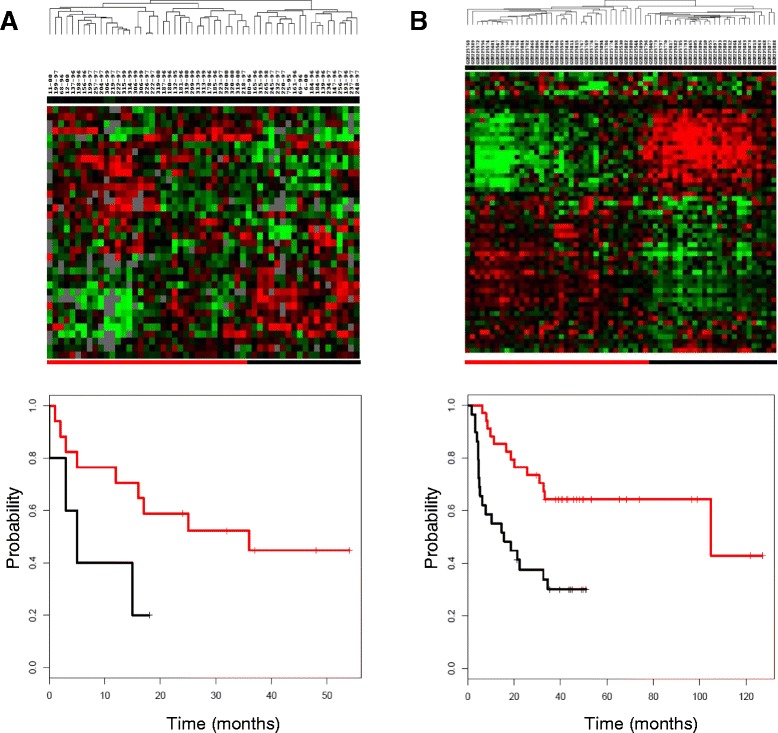
Prognostic significance of the “Fibroblast specific BMP2 induced gene list” in human lung adenocarcinomas. Unsupervised hierarchical clustering of the genes in the “Fibroblast specific BMP2 induced gene list” in the Garber and the Lee datasets. **(A)** The expression values of genes in the “Fibroblast specific BMP2 induced gene list” were extracted from a published study of genes expressed in 41 lung adenocarcinomas. The genes are presented in rows and experiments, in columns. The “Fibroblast specific BMP2 induced gene list” stratifies lung adenocarcinomas into two groups. The Kaplan-Meier curve on the lower panel represents the clinical outcome of the patients with tumors exhibiting high (underlined in black) and low expression levels (underlined in red) of genes in the “Fibroblast specific BMP2 induced gene list”. **(B)** Expression of the “Fibroblast specific BMP2 induced gene list” in stage I lung adenocarcinomas published by Lee et al. Again, the “Fibroblast specific BMP2 induced gene list” segregates lung adenocarcinomas into two groups which have significantly different clinical outcomes as shown by the Kaplan-Meier curves of overall survival.

To verify the relevance of our *in vitro* experiments, we checked the expression of genes in the “Fibroblast specific BMP2 induced gene list” (Additional file 7: Table S7) in publicly available microarray data from lung cancer biopsies [13]. Garber et al. published global gene expression profiles of 41 human lung adenocarcinomas; patient survival data were available for 24 of these adenocarcinomas [13] (GEO: GSE3398). A subset of the genes that overlapped between the “Fibroblast specific BMP2 induced gene list” and the Garber dataset (n = 37), was coherent to provide a basis for segregation of the tumors into two groups (Figure 5A). As visualized in the Kaplan-Meier plots, the lung cancer patients with a high expression level of the “Fibroblast specific BMP2 induced gene list” showed a strong trend to a higher risk of death than the patients with a low expression level (76% versus 38% after 12 months, hazard ratio (HR): 0.57, cox-p = 0.07). Thus, the “Fibroblast specific BMP2 induced gene list” has a potential to be a prognostic marker in lung cancer.

To further support this finding we analyzed the stage I lung adenocarcinomas published by Lee et al. [14]. From 67 genes building “Fibroblast specific BMP2 induced gene list” 48 were present in Lee dataset. Patients stratification based on unsupervised hierarchical clustering divided patients into two groups. Patients carrying tumors with high expression levels of the “Fibroblast specific BMP2 induced gene list” showed a significantly higher risk of death than the patients with a low expression level (76% versus 38% after 24 months, hazard ratio (HR): 0.49, cox-p =0.002) (Figure 5B).

**Figure 6:**
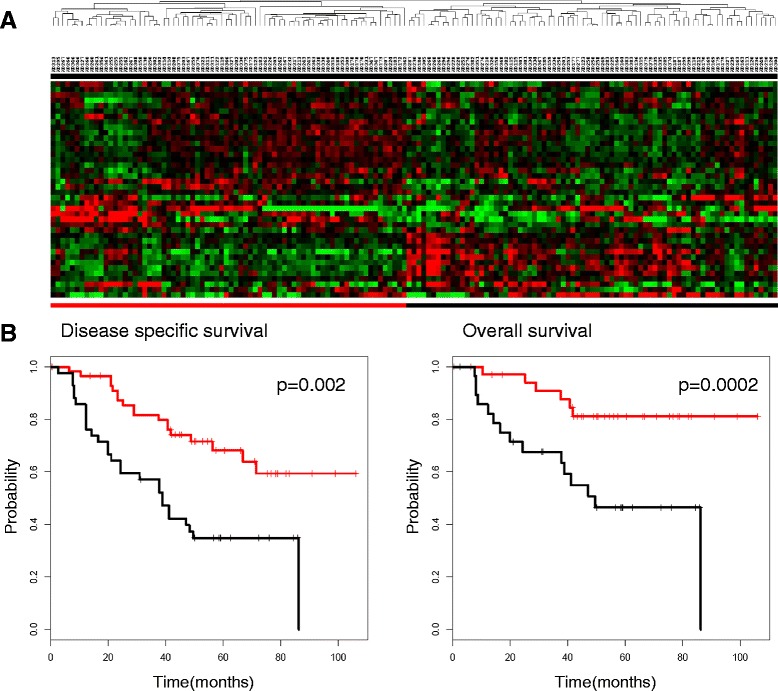
Prognostic significance of the “Fibroblast specific BMP2 induced gene list” in human stage I lung adenocarcinomas. **(A)** Unsupervised hierarchical clustering of the “Fibroblast specific BMP2 induced gene list” in the Bhattacharjee dataset. The expression values of the genes in the “Fibroblast specific BMP2 induced gene list” were extracted from a published expression study of 151 stage I lung adenocarcinomas. The “Fibroblast specific BMP2 induced gene list” stratifies lung adenocarcinomas into two groups. **(B)** The relationship between the expression levels of genes comprising the “Fibroblast specific BMP2 induced gene list” and the overall and disease-specific survival. The Kaplan-Meier curves represent the clinical outcomes of the patients.

We further validated our findings using a larger and better-annotated dataset published by Bhattacharjee [15], which contains microarray profiles of 151 stage I lung adenocarcinomas from patients who had undergone surgery. Consistent with our hypothesis, based on 66 genes present in the dataset, the expression of genes of the “Fibroblast specific BMP2 induced gene list” provided a basis for segregation of the tumors into two groups (Figure 6). Compared with patients with low expression levels of genes in the “Fibroblast specific BMP2 induced gene list” (underlined in red), patients with high expression levels of these genes (underlined in black) had a significantly shorter disease-specific survival (87% versus 46% after 5 years, HR: 0.57, cox-p = 0.002) and overall survival (68% versus 34% after 5 years, HR: 0.51, cox-p = 0.0002). Both Kaplan-Meier curves are shown in Figure 6.

**Figure 7:**
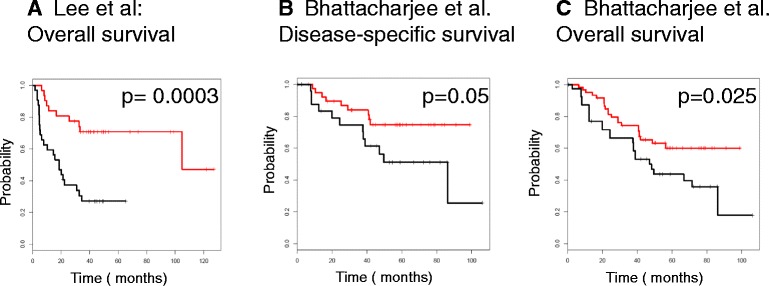
Survival analysis of stage I lung adenocarcinomas using continuous scoring of the Lee et al. and the Bhattacharjee et al. datasets. The relationship between the expression levels of genes comprising the “Fibroblast specific BMP2 induced gene list” and the overall and disease-specific survival based on continuous scoring as described in the methods section. The Kaplan-Meier curves for **(A)** overall survival in the Lee et al. dataset and **(B)** disease specific survival and **(C)** overall survival in the Bhattacharjee et al. dataset are presented. The survival-curves of patients with down-regulated centroid of the “Fibroblast specific BMP2 induced gene list” are encoded in red, with up-regulated centroid in black.

Because the classification of data based on hierarchical clustering has been suggested to be unstable and codependent on many factors such as the presence of missing values [47], we validated the results using continuous scoring and stratified the patients in the Lee and the Bhattacharjee datasets based on a score derived from the average expression level of genes in the “Fibroblast specific BMP2 induced gene list”. The continuous scoring approach consistently divided the lung cancer patients into two groups with significantly different outcomes (Lee dataset: overall survival: HR 0.38, cox-p = 0.00028, Bhattacharjee dataset: disease free survival: HR 0.69, cox-p = 0.0023, overall survival: HR 0.72, cox-p =0.027) (Figure 7). Taken together, these findings indicate that genes included in the “Fibroblast specific BMP2 induced gene list” are of importance *in vivo* in human lung carcinomas and helpful in predicting outcomes for patients with lung cancer.

## Discussion

Gene expression signatures have been described in a number of studies as surrogate phenotypes to explore the relevance of *in vitro* findings *in vivo* in human cancer [20-22]. In this study, we have taken this approach to explore the effects of BMP stimulation of fibroblastic stroma *in vitro* on lung cancer. It has long been established that epithelial-mesenchymal interactions are essential for lung development and organogenesis [23]. Given the multifaceted roles of BMP signaling in these processes [24,25], investigating its transcriptional effects on fibroblastic stromal cells might improve our understanding of lung carcinogenesis. In this study we provide a comprehensive genomic analysis of gene expression changes in lung fibroblasts in response to stimulation with BMP2, BMP4, BMP7 and their antagonists Gremlin and Noggin. Our results reveal alterations in the expression of a diverse spectrum of genes, reflecting changes in various components of key signaling pathways involved in tumorigenesis.

Stimulation of primary human lung fibroblasts with BMP2 significantly changes their gene expression pattern through signaling via the BMP pathway, as shown by changes in the expression of genes in the BMP pathway and BMP target genes, such as BMPR2A, BAMBI, SMAD7ID1, ID2, and ID3. Of specific interest is the induction of TPM1, TPM2, TNC and POSTN, suggesting a fundamental reprogramming of lung fibroblasts such that the cells exhibit a gene expression profile that is typical for carcinoma-associated fibroblasts. Of specific interest is also the expression of genes, which might support cancer stem cells by forming a stem cell niche. POSTN e.g. has recently been shown to be an essential component of the metastatic stem cell niche [26]. Preparation of the metastasis location is an essential step in the cascade of effects leading to the development of fatal metastatic disease. It would be of great interest to further investigate and clarify the role of BMPs in the lung cancer stem cell niche. Furthermore as a result of the GO-term analysis, the mRNA expression levels of GATA6, ID2, AHR, IGFBP7, BAMBI, SERPINE1, SMAD6, TRNP1, and CYR61 increased in response to BMP2 stimulation, suggest that BMP2 stimulation of fibroblasts plays a role in the regulation of cell proliferation. GATA6, DKK1, ID2, BAMBI, SERPINE1, CYR61 and PTGIS indicate a positive regulation of a developmental process. The local regulation of diverse cell signaling pathways by BMP in fibroblasts could therefore have multifaceted consequences for tissue microenvironments *in vivo*, including the balance between proliferation and development. The interconnectivity of these regulatory systems may contribute to the robustness of stem cell niches and the precise spatial and temporal control of differentiation.

To clarify the *in vivo* relevance of our *in vitro* finding of an activation of the fibroblastic stroma, we correlated the expression of the “fibroblast specific BMP2 induced gene list” with the outcome of lung adenocarcinoma biopsies. Up-regulation of the “fibroblast specific BMP2 induced gene list” is associated with a significantly worse disease-free and overall survival as shown in two datasets of operated stage I lung adenocarcinoma patients, and one set of human lung adenocarcinoma biopsies.

Since segregation of patient samples using unsupervised hierarchical clustering might be unstable and dependent on missing values, we confirmed the finding using a more robust approach based on a score derived from the mean expression values (centroid) of all the genes in the signature. This suggests that the “fibroblast specific BMP2 induced gene list” is prognostic in lung adenocarcinomas. This hints to the *in vivo* biological relevance of our *in vitro* finding of an up-regulation of this gene list by BMP2 stimulation in lung fibroblasts. Of note this does not necessarily mean that any gene subsets or single genes of the “fibroblast specific BMP2 induced gene list” have the same prognostic power, neither it excludes existence of other, parallel prognostic signatures.

In line with our findings, BMP2 has been shown to be over-expressed in lung cancer [2]. BMP2 serum levels have been shown to be higher in lung cancer patients than in normal healthy controls [27]. There was a positive correlation of tumor burden and tumor stage. Not surprisingly BMP2 serum levels were correlated with patient outcome [28]. In our analysis the effect of BMP2 stimulation expressed by the “fibroblast specific BMP2 induced gene list” is correlated with patient outcome even in the same tumor stage, namely stage I tumors. This hints to a prognostic relevance of the BMP2 effect in lung cancer, which is independent from stage and tumor burden. Further *in vitro* evidence suggests an essential role of BMP2 in lung cancer. Knockdown experiments of BMP2 in A549 lung adenocarcinoma cells had shown decreased growth and invasion potential by changes in the tumor cells gene expression pattern revealing therapeutic targets and strategies [29].

Our analysis of the changes in the expression pattern of the stromal fibroblasts upon BMP2 stimulation adds additional insights in tumorigenesis and potential therapeutic targets. Stromal-targeted therapy in lung cancer has not yet become a clinical reality due to several challenges. Identification of tumorigenic mechanisms and factors, as e.g. BMP signaling in stromal cells, is a prerequisite for the design of potential novel therapeutic strategies.

## Conclusion

Bone morphogenetic proteins play important roles in development, morphogenesis and cancer. We showed that the effects of BMPs and their antagonists, gremlin and noggin, are heterogeneous in different cell types. The gene expression pattern induced by BMP2 in lung fibroblasts significantly correlated with the prognosis of patients with stage I lung adenocarcinomas.

## Additional files

Additional file 1: Table S1. Table of genes with a change in expression level greater than 1.5-fold after stimulation with BMP2 are color coded. 171 genes induced by BMP2 stimulation are encoded in red (mean fold up-regulation 2.68 ± 0.86), 206 genes repressed by BMP2 stimulation in green (mean fold down-regulation 2.99 ± 1.604). Additional file 2: Table S2. List of the genes building the “CCL-171 derived BMP2 signature”. Additional file 3: Table S3. GO Term Analysis: The analysis revealed that the ”CCL-171-derived BMP2 signature” is significantly enriched for genes involved in biological processes such as the cellular response to chemical stimuli and the BMP signaling pathway with Bonferoni corrected p-values of 0.00017 and 0.006, respectively. Additional file 4: Table S4. Biologically independent duplicated samples of CCL-171 cells were stimulated with BMP2 and the gene expression profile assessed with HEEBO microarrays. After filtering for data quallity as described in the methods section, a two-class unpaired significance analysis of microarrays was performed on 13601 genes. Genes with a false discovery rate <1% were selected. 76 genes were significantly induced and 151 genes repressed by BMP2 stimulation of CCL-171 cells. Additional file 5: Table S5. 115 genes of the “common BMP-induced gene list”. Additional file 6: Table S6 SAM results of genes commonly induced and repressed by BMP2. SAM on 13770 genes after filtering as described in methods section have been submitted to a multiclass unpaired SAM with block permuation. Genes with a FDR < 1% were selected. 80 genes were significantly induced and 200 significantly repressed by BMP2 in CCL-171, MDA-MB-231 and T47D. Additional file 7: Table S7. List of the genes building the “Fibroblast specific BMP2 induced gene list” as indicated in the Figure 4.
